# Vaccination and Platelet Biology: Unraveling the Immuno-Hemostatic Interplay

**DOI:** 10.3390/vaccines13040403

**Published:** 2025-04-13

**Authors:** Sneha Ratnapriya, Shivraj M. Yabaji

**Affiliations:** 1 Center for Immunology and Inflammatory Diseases, Massachusetts General Hospital, Harvard Medical School, Boston, MA 02114, USA; 2 The National Emerging Infectious Diseases Laboratory, Boston University, Boston, MA 02215, USA

**Keywords:** platelets, innate immunity, adaptive immunity, vaccine, granules, cytokines, vaccine-induced immune thrombotic thrombocytopenia

## Abstract

Platelets, which have been traditionally associated with hemostasis and thrombosis functions, now receive attention for their role in immune responses that may affect vaccine development and effectiveness. Through their interactions with immune cells and modulation of inflammation alongside their role in antigen presentation, platelets become integral components of both innate and adaptive immune systems. New research shows platelets can improve vaccine effectiveness while reducing adverse side effects. During vaccine administration, platelets release cytokines and chemokines, which attract and stimulate immune cells to the injection site. Platelets work together with dendritic cells and T cells to support antigen processing and presentation, which leads to strong immune activation. Platelets’ pro-inflammatory mediators strengthen local immune responses to boost protective immunity generation. Significant attention has been given to platelet involvement in vaccine-related thrombotic events, including vaccine-induced immune thrombotic thrombocytopenia (VITT). The rarity and severity of these events demonstrate the need to investigate the complex interplay between vaccine mechanisms and platelet activation. Exploration of the platelet-immune axis can lead to new methods for improving both the effectiveness and safety of vaccines. Researchers are working on creating innovative approaches for treatments that target platelet receptors and thrombosis pathways without interfering with the regular hemostatic functions of platelets. New vaccine development methods and personalized immunization strategies can emerge from targeting platelets with adjuvants and immune modulators.

## 1. Introduction

Platelets are small, non-nucleated, discoid-shaped blood components produced from megakaryocytes in the bone marrow where erythrocytes (red blood cells) and most leukocytes (white blood cells) [1] are also formed. Megakaryocytes are large bone marrow cells, and they mature and fragment to release over 1000 platelets per megakaryocyte. The key hormone regulating megakaryocyte development is thrombopoietin (TPO). Platelets have a diameter of 2–3 μm and a 7–10 day lifespan in circulation [2]. Despite lacking a nucleus, they are equipped with a complex molecular machinery that enables their participation in immune responses [3]. Platelets possess a cytoskeleton composed of actin filaments and microtubules, which allow shape changes during activation [4]. Their plasma membrane is enriched with receptors such as glycoproteins (e.g., GPVI and GPIbα) and integrins (e.g., αIIbβ3 or GPIIbIIIa) that facilitate interactions with extracellular matrix proteins and immune cells [5].

Platelets contain three major types of granules which store a wide array of bioactive molecules critical for all the immune functions performed by platelets. These granules are described below and in Table 1.

### 1.1. Alpha Granules

Alpha (α) granules are the most abundant platelet granules, with 50–80 per platelet. Their formation in megakaryocytes involves endosomal pathways and multivesicular bodies, relying on newly synthesized cargos from the trans-Golgi network (TGN) and endocytosed proteins, VPS33B, VPS16B, and NBEAL2 [6,7]. These large, membrane-bound structures store proteins essential for coagulation, inflammation, and tissue repair [8]. Alpha (α) granules contain adhesion molecules like P-selectin, von Willebrand factor (vWF), and fibrinogen, which facilitate platelet aggregation and interactions with endothelial cells and leukocytes [9]. They also release growth factors such as VEGF, PDGF, and TGF-β, supporting tissue repair, angiogenesis, and immune modulation [10]. Additionally, cytokines and chemokines, including IL-1β, CXCL4, and RANTES, contributing to inflammatory processes are also present in the granules [10].

Some α granule proteins are synthesized by megakaryocytes, while others, like fibrinogen and Factor V, are endocytosed from circulation [11]. Upon platelet activation, α granules release their contents in a tightly regulated manner [12]. P-selectin is translocated to the surface, enhancing leukocyte recruitment and adhesion, thereby amplifying the inflammatory response. The released proteins also aid in tissue repair and inflammation resolution, highlighting α granules’ vital role in hemostasis and immune regulation.

### 1.2. Dense Granules

Dense granules are smaller in size and less numerous than α granules, with an average of 3–8 granules per platelet [6]. DG formation involves cargo sorting by the AP-3 complex, stabilized by protein complexes, BLOC-1, with BLOC-2 and BLOC-3 suspected to function downstream. Rab38/Rab32 facilitate cargo trafficking to mature DGs, aided by BLOC-3, which serves as a guanine nucleotide exchange factor for these Rabs [13]. These granules contain low molecular weight compounds, including adenosine diphosphate (ADP) and adenosine triphosphate (ATP), which amplify platelet activation and recruit additional platelets to the site of injury, calcium ions (Ca^2+^), which are essential for platelet activation and coagulation cascade signaling, and small molecules like serotonin, a vasoactive molecule that modulates vascular tone and permeability. These also contain polyphosphates that enhance coagulation and support innate immune responses [11]. Dense granules are released rapidly upon platelet activation and likely contribute to the recruitment and activation of several immune cells such as neutrophils and monocytes [14]. Their contents also play a role in promoting vasodilation and increasing vascular permeability, facilitating the extravasation of immune cells to sites of injury, infection, or inflammation [15].

### 1.3. Lysosomes

Lysosomes in platelets contain hydrolytic enzymes, including proteases, glycosidases, and lipases [9]. These enzymes are primarily involved in degrading extracellular matrix components and removing damaged or infected tissues [9]. While lysosomes are less studied than alpha and dense granules, their role in immune responses and tissue remodeling is increasingly recognized.

The regulation of the granules’ release is crucial for the platelets’ function. Platelet secretion may need to be differential, especially when comparing the release of dense granule and α-granule cargo, but it remains unclear whether this is required for specific platelet functions at injury sites [16]. Further analysis is needed to understand whether platelet secretion is controlled by differences in release kinetics, differential solubility of cargo proteins, or other mechanisms, as platelets may have more complexities to reveal in their biology [17].

Italiano et al. proposed that platelets selectively store and release granular cargo based on specific physiological needs, such as promoting angiogenesis by releasing VEGF or inhibiting it by releasing angiostatin from distinct granule subsets [18]. This idea stems from two key observations: (1) Pro- and anti-angiogenic factors were localized in separate compartments; (2) Ma et al. demonstrated that different agonists (e.g., PAR1 and PAR4 peptides) triggered the selective release of specific cargo proteins. This hypothesis has gained significant attention, suggesting that platelets function as “smart” delivery systems capable of context-dependent cargo release [19,20]. While the hypothesis of differential granule exocytosis positions platelets as highly adaptable effector cells, questions remain about the extent of α granule diversity and whether the secretory machinery in platelets is sophisticated enough to enable this level of selective cargo release. Further research is required to fully understand these mechanisms and their physiological relevance.

## 2. Multifunctional Roles of Platelets in Human Physiology

**Figure 1:**
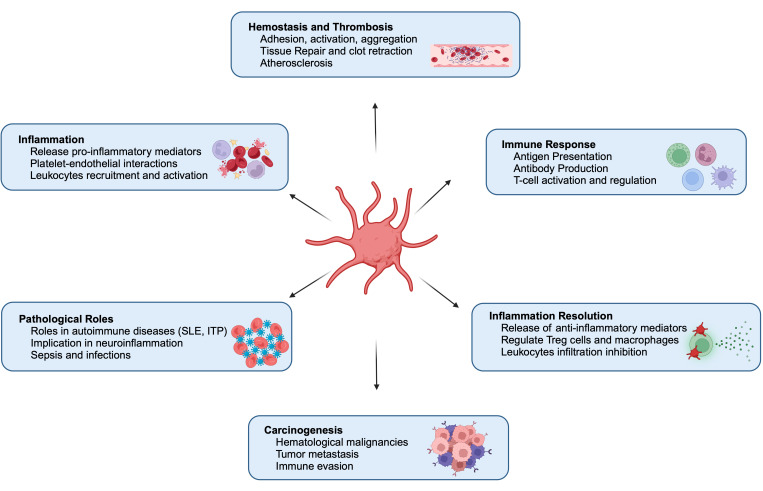
Multifaceted roles of platelets in health and diseases [1,2,3,10,11,17,21,22,23,24,25,26,27,28,29] (created in BioRender. Yabaji, S. (2025) https://BioRender.com/dioo3wt, accessed on 8 April 2025).

Platelets, traditionally known as small cellular fragments circulating in blood and assisting in hemostasis and thrombosis, are being acknowledged as dynamic immune cells. These small anucleate components of the blood are not merely bystanders in the immune system but active participants that influence innate and adaptive immunity [21]. Platelet endorsement as immune sentinels and mediators of immune surveillance and vascular remodeling has shifted our understanding of platelets, leading to profound implications for immunology. Platelets also have anti-inflammatory potential by interacting with regulatory T cells (Tregs), macrophages, and leukocytes and releasing pro-resolving mediators (ResolvinE1, Lipoxin, and Maresin1) (Figure 1). Thus, the dual nature of platelets as both pro-inflammatory and anti-inflammatory entities underscores their complexity and adaptability in various physiological and pathological contexts [22].

Platelet activation is essential for hemostasis, immune regulation, and various physiological functions (Figure 1). These can be grouped into the following key areas:

**Hemostasis and Thrombosis**: Activated platelets adhere to vascular injury sites, change shape, release granule contents, and aggregate to form a hemostatic plug, preventing excessive bleeding and maintaining vascular integrity. Dysregulated activation can lead to thrombosis, causing vessel occlusion and events like heart attacks or strokes [23].

**Inflammatory Responses**: Platelets interact with leukocytes and endothelial cells, recruiting inflammatory cells to injury or infection sites. They release cytokines, chemokines, and growth factors that drive immune cell function, inflammation, tissue repair, and regeneration [24].

**Angiogenesis**: Platelets release factors like VEGF and PDGF that stimulate endothelial cell growth, migration, and new blood vessel formation, crucial for tissue repair and regeneration [25].

**Wound Healing**: Platelet-derived growth factors (e.g., TGF-β and FGF) promote cell proliferation, the formation of an extracellular matrix, and tissue remodeling, which are essential for healing and wound closure [30].

**Immune Modulation**: Platelets modulate immune responses by interacting with immune cells, influencing cytokine production, and presenting antigens to T cells. This contributes to immune activation and regulation [26].

**Vascular Function**: Platelets release vasoactive substances like serotonin and thromboxane A2, inducing vasoconstriction and aggregation. They also interact with endothelial cells to regulate vascular tone, permeability, and inflammation [27,31].

Overall, platelet activation effects are diverse and multifaceted, constituting their primary roles in hemostasis and inflammation (Figure 1). They also assist in angiogenesis and immune modulation. Understanding and addressing these functions is essential for unraveling the complex roles of platelets in health and different infectious and inflammatory situations, such as cardiovascular disease and autoimmune disorders, and developing targeted therapeutic interventions for platelet-related disorders.

## 3. Synergy of Platelets and Macrophages in Immune Regulation

Platelets and macrophages synergize to perform immunomodulatory functions by regulating innate immune response and inflammation (Figure 1). This interaction enhances the inflammatory response and facilitates pathogen clearance. Activated platelets release a variety of cytokines, chemokines, and growth factors, such as platelet factor 4 (PF4) and transforming growth factor-beta (TGF-β), which recruit and activate macrophages at sites of injury or infection [27,31,32,33,34]. Macrophages, in turn, respond by releasing pro-inflammatory cytokines like tumor necrosis factor-alpha (TNF-α) and interleukin-6 (IL-6), further amplifying the inflammatory cascade [21,35,36,37,38,39].

Moreover, platelets significantly influence macrophage polarization during inflammation, infection, and injury, guiding them toward either the pro-inflammatory M1 phenotype or the anti-inflammatory M2 phenotype based on specific environmental cues [28,40,41,42,43]. This dynamic interaction contributes to various physiological and pathological processes, including tissue repair, chronic inflammation, and thrombo-inflammatory disorders. For instance, platelet-derived epidermal growth factor receptor (PDGFR) has been shown to induce M1 macrophage polarization by increasing the expression of inducible nitric oxide synthase (iNOS) and CD64, thereby enhancing pro-inflammatory responses and bacterial clearance [44,45]. Conversely, platelet-rich plasma (PRP), which is abundant in anti-inflammatory mediators and growth factors, has been observed to suppress M1 polarization and promote M2 polarization, facilitating tissue repair and reducing inflammation [46]. This dual capacity highlights the pivotal role of platelets in immune response modulation across various physiological and pathological contexts. The complex interplay between platelets and macrophages highlights the context-dependent effects of platelets on macrophage polarization and the subsequent implications for immune responses and tissue homeostasis.

However, dysregulated platelet–macrophage interactions can exacerbate conditions like atherosclerosis and autoimmune diseases, emphasizing the need for targeted therapies to modulate this synergy. Understanding the mechanisms underlying platelet–macrophage communication provides insights into novel treatment strategies for inflammation-driven diseases [21,27,34,37].

## 4. Platelets as Mediators of Innate Immunity

Platelets play an essential role in innate immunity by acting as sentinels that recognize and respond to pathogens (Figure 1). Neutrophils are key innate immune responders, and platelet-derived factors are crucial in their recruitment. P-selectin and the serotonin metabolite, 5-Hydroxy indoleacetic acid (5-HIAA) promote neutrophil migration via PSGL1 and GPR35, respectively. Platelet TLR4, p110β, and CLEC-2 signaling further regulate neutrophil infiltration in inflammation, tissue repair, and anti-tumor responses, underscoring the importance of platelets in neutrophil mobilization [19,20,47,48,49,50].

Platelets express key pattern recognition receptors (PRRs), including Toll-like receptors (TLRs), C-type lectin receptors (CLRs) such as DC-SIGN, and NOD-like receptors (NLRs). They detect bacterial pathogens via TLR4, triggering neutrophil activation and NETosis (formation of neutrophil extracellular traps) without inducing classical aggregation [51]. Unlike nucleated immune cells, platelet TLR4 signaling may function independently of Myd88. In murine *Klebsiella pneumoniae* infection models, platelet-specific Myd88 deficiency did not affect outcomes, suggesting alternative pathways [52]. Platelet NOD2 recognizes muramyl dipeptide (MDP) and signals via Rip2, influencing sepsis responses and thrombosis [53]. TLR4 activation also drives IL-1β-rich microparticle release, enhancing neutrophil interactions and NET formation, which helps trap bacteria and modulate immune responses [51,54].

Platelets are a major source of IL-1β, which is not stored in granules but produced upon stimulation. Surprisingly, platelets express pre-mRNA for IL-1β, which is spliced and translated into pro-IL-1β, then processed by caspase-1 to release functional IL-1β over several hours. In a mouse model of severe malaria, platelet-derived IL-1β plays a critical role in initiating the acute phase response, with platelets localized to hepatic sinusoids post-plasmodium infection, suggesting a contact-dependent mechanism [55,56].

Platelets express FcγRIIa, a low-affinity receptor for monomeric IgG, which binds immune complexes, IgG-coated pathogens, and autoantibodies targeting platelet proteins [57,58], facilitating interactions with Influenza (H1N1), Streptococci, and Bacillus anthracis. FcγRIIA is key in heparin-induced thrombocytopenia (HIT), recognizing anti-PF4/heparin complexes and activating platelets via Src/Syk signaling, leading to thrombosis [59,60,61,62]. It also interacts with αIIbβ3 integrin, transmitting “outside-in” signals essential for full platelet activation [57]. Platelets also express CLEC-2, which binds podoplanin to regulate blood-lymphatic vessel separation. In sepsis, it promotes an anti-inflammatory macrophage phenotype, while in *Salmonella typhimurium* infection, hepatic podoplanin upregulation activates CLEC-2, driving thrombus formation. These findings highlight CLEC-2’s dual role in inflammation and thrombosis [63,64].

Platelets engage innate immune pathways for host defense. The stimulator of interferon genes (STING) regulates their activity via STXBP2, promoting granule release through SNARE complex formation. STING deficiency in platelets reduces NET formation and thrombosis in sepsis, with similar protective effects observed with blocking STING-STXBP2 interactions [65,66]. Platelets also express the NLRP3 inflammasome, activating Caspase-1 to process IL-1β, and use the cGAS (cyclic GMP-AMP synthase)-STING pathway to amplify antiviral and inflammatory responses [44,67]. Although anucleated, platelets release mitochondria upon activation, triggering inflammation through pro-inflammatory lipids and mitochondrial DNA (mtDNA). The mtDNA stimulates thrombin generation and neutrophil activation, contributing to immunopathology in SLE via FcγRIIa. Platelet microparticles (PMPs) further influence inflammation and access immune sites, though their precise role in host defense remains unclear [68,69,70,71,72,73].

## 5. Platelets as Adaptive Immune Regulators

Host defense involves a multi-layered process, where innate immune cells take on all intruders providing immediate protection, followed by T and B lymphocytes coming into play ensuring specific and long-lasting responses or adaptive immunity [74]. While adaptive immunity takes days to develop, it is essential for long-term specific protection. Platelets play a significant role in adaptive immunity (Figure 1), influencing antigen trafficking and presentation by dendritic cells (DCs) and modulating T and B cell signaling, maturation, and polarization [75]. Platelets affect adaptive immune cells by activating monocytes, macrophages, T and B cells, DCs, and NK cells [76].

Lymph nodes are crucial for antigen presentation and lymphocyte trafficking [77], with CLEC-2 expressed by platelets playing a key role in their development and maintenance. Platelet-specific CLEC-2 deletion leads to blood-filled lymph nodes, fibrosis, and impaired antibody production [78]. Beyond supporting lymph node function for effective adaptive immunity, platelets also directly influence antigen-presenting dendritic cells (DCs) [76]. They promote DC differentiation via the P-selectin-PSGL-1 axis and enhance type I interferon production through CD40L [79]. CD40L-driven DC activation and interferon production contribute to autoimmunity in lupus [80], where innate immune activation and coagulation crosstalk are amplified by endothelial protein C receptor (EPCR) and tissue factor signaling.

CLEC-2, expressed by platelets, plays a key role in the lymph nodes’ development and maintenance, the deletion of which leads to blood-filled lymph nodes, fibrosis, and impaired antibody production [63,81]. Platelets also promote dendritic cell differentiation via the P-selectin-PSGL-1 axis and enhance type I interferon production through CD40L [79]. Platelets exert diverse effects on lymphocyte subpopulations. They can suppress cytokine secretion and immunosuppressive responses of T helper cells while enhancing cytotoxic T cell proliferation and activity. Activated platelets can promote B cell isotype switching and antibody production but reduce the cytolytic activity of natural killer (NK) cells [82].

## 6. Platelets in Vaccine-Induced Immune Thrombotic Thrombocytopenia

Platelets play a critical role as effector cells in adverse immune responses to vaccines, with a notable example being vaccine-induced immune thrombotic thrombocytopenia (VITT) [83]. For most individuals, administering an adenovirus-based COVID-19 vaccine effectively triggers the production of antibodies against the SARS-CoV-2 spike protein, thereby providing strong protection against COVID-19 [84]. However, in rare cases, the vaccine can induce VITT. Platelet-activating antibodies targeting platelet factor 4 (PF4), a platelet-associated chemokine, trigger this condition. These antibodies form immune complexes with PF4, which are then recognized by platelet FcγRIIa receptors, leading to pathological platelet activation, initiating a cascade of coagulating events, leading to blood clots or thrombosis and thrombocytopenia or reduction in platelet count [85]. While VITT represents a severe pathological reaction, platelets may also exert an intended immune response upon SARS-CoV-2 vaccination [86], probably programmed cell death pathways activation to control viral replication. This raises an intriguing question about how platelets respond to the vaccine and potentially contribute to effective immunization, which underscores the importance of understanding immune responses to vaccination.

Von Willebrand factor (vWF), a multimeric adhesive protein, plays a role in the formation of platelet-rich thrombi in VITT and various thrombotic and hemorrhagic conditions [29]. The vWF protein attaches to extracellular DNA released by neutrophils, forming NETs, which promote neutrophil adhesion to the endothelium [85,87]. The vWF forms a complex with PF4, recognized by anti-PF4 antibodies, leading to the generation of immune complexes and ultimately activating platelets [88]. NETs contribute to thrombosis by providing a structural framework that facilitates the adhesion of platelets and platelet-binding ligands, such as vWF and fibrinogen [89]. NETosis, triggered by platelet interactions with anti-PF4 antibodies, depends on neutrophil–platelet binding through PSGL-1 and P-selectin. Blocking these interactions diminishes NETosis and the release of neutrophil DNA [12]. This underscores the essential role of platelet-neutrophil interactions in promoting clot formation during NETosis in VITT. Another important mechanism contributing to VITT is the activation of the complement system, which has been associated with immune-mediated platelet dysfunction and thromboinflammation in conditions like anti-phospholipid syndrome [90]. Complement activation intensifies platelet activation and aggregation in VITT through various mechanisms. One pathway involves the formation of PF4 immune complexes with vaccine components, such as adenoviral capsid proteins or DNA, ultimately leading to the activation of C3 and the subsequent generation of C3a, C5a, and the membrane attack complex on platelets, contributing to vascular damage and exacerbating the prothrombotic environment [91]. Complement activation in VITT also fosters a feedback loop or self-enhancing activation cascade, where platelet activation further stimulates the complement system, worsening inflammation and coagulation [92]. Activated complement fragments drive thromboinflammatory responses by engaging complement receptors on platelets, monocytes, and neutrophils.

In the review by Hirsch et al., they effectively explore the interaction between platelets and neutrophils, with a particular focus on COVID-19 and VITT [93]. They highlight the key roles of hyperactivated platelets and NETs in the coagulopathy seen in COVID-19 and discuss the development of a new thrombophilia paradigm triggered by auto-antibody formation following adenoviral vector vaccinations [93]. It was reported that pulmonary embolism and thrombosis in many COVID-19 patients are associated with elevated P-selectin expression on endothelial cells, which enhances platelet aggregation and clot formation [94,95]. This finding points to the importance of endothelial cell activation in vaccine-induced thrombotic events. Understanding the role of P-selectin in platelet–endothelium interactions may provide further insights into VITT [85]. In another study by Ostrowski et al., a direct comparison between the Oxford/AstraZeneca [ChAdOx1] (AZ) and mRNA vaccines was carried out that revealed enhanced inflammation, platelet activation, and thrombin generation following AZ vaccination, further supporting potential triggers and mechanisms underlying complications like VITT [96].

Rapid treatment is essential for VITT patients, as delays can be life-threatening. Since FcγRIIa mediates VITT, immune-suppressive therapies are effective. The American Society of Hematology recommends intravenous immunoglobulin (IVIG) as the first-line treatment at 1 g/kg daily for two days, often supplemented with corticosteroids like dexamethasone. Studies have shown that IVIG significantly reduces mortality in VITT patients, validating its use as a key treatment strategy [97,98,99]. Targeting fibrinolysis may offer a promising treatment for VITT. Enhancing fibrinolysis has shown effectiveness in a mouse model of HIT [100].

## 7. Therapeutic Implications of Platelets

Beyond their usual physiological and pathological functions, platelets offer significant therapeutic potential in many clinical applications. One of the primary therapeutic uses of platelets is in transfusion medicine [101]. Platelet transfusions are essential for patients with thrombocytopenia or platelet dysfunction to prevent or control bleeding. In oncology, platelet transfusions are critical for managing bleeding risks in patients undergoing chemotherapy or bone marrow transplantation [102]. Platelets also hold promise in regenerative medicine and wound healing. Platelet-rich plasma (PRP), derived from concentrated platelets, is widely used to promote tissue repair and regeneration [103]. Growth factors such as platelet-derived growth factor (PDGF) and vascular endothelial growth factor (VEGF) in PRP stimulate cell proliferation, angiogenesis, and extracellular matrix formation [103], making it valuable for treating sports injuries, chronic wounds, and osteoarthritis. However, although platelet transfusions may be considered in select cases, they are generally reserved for patients with active bleeding, as transfused platelets are often functionally compromised and have a short circulation time of 6–24 h.

Antiplatelet drugs are the primary treatment for cardiovascular disease and atherothrombosis prevention [103]. Current therapies have limitations, with few drugs available for managing platelet-related bleeding. Treatments are mostly restricted to increasing platelet count (e.g., thrombopoietin) or platelet transfusions for conditions like thrombocytopenia [104]. Aspirin (acetylsalicylic acid) is the most commonly prescribed drug for cardiovascular disease, working by irreversibly inhibiting cyclooxygenase-1 and blocking thromboxane A2 formation [105]. Despite an associated bleeding risk, low-dose aspirin is recommended for primary prevention in individuals at high cardiovascular risk. For secondary prevention in acute coronary syndrome or post-coronary stent implantation, dual antiplatelet therapy (DAPT), combining aspirin with P2Y12 receptor blockers, is the standard [106]. Irreversible P2Y12 inhibitors include clopidogrel and prasugrel, while ticagrelor and cangrelor provide faster, reversible effects. For symptomatic peripheral artery disease, single antiplatelet therapy with aspirin or clopidogrel is preferred due to a better benefit–risk ratio compared to DAPT, although the risk of recurrent thrombosis remains significant in these patients [107,108,109,110].

The relevance of platelet immunology extends beyond basic science to the realm of drug development and therapeutics. Platelets represent both a target and a tool in the quest to modulate immune responses. Antiplatelet therapies, traditionally used to prevent thrombosis, are being repurposed to mitigate inflammation in autoimmune and cardiovascular diseases. Drugs such as aspirin and P2Y12 inhibitors block TXA2 formation and inhibit ADP agonist receptors, respectively, [111] (Table 2), causing inhibition of platelet activation, and have shown promise in reducing immune-mediated vascular damage and improving outcomes in inflammatory diseases. In addition, platelet-derived biomarkers such as proteins and RNA present inside the platelets are emerging as valuable tools for cancer diagnosis, and monitoring and confronting immune-related inflammatory conditions [112]. Platelet microparticles, cytokines, and extracellular vesicles provide insights into disease activity and therapeutic efficacy, paving the way for personalized medicine.

Recent advancements in platelet biology highlight their potential to secrete several pro- and anti-inflammatory mediators that modulate immune functions, which could be further utilized for designing and developing therapies for autoimmune diseases and inflammatory conditions. Additionally, emerging evidence suggests targeting platelet activation pathways for treating thrombotic disorders, such as heart attacks and strokes [113,114,115,116]. Overall, platelets are a versatile tool in therapeutic interventions, with ongoing research expanding their applications in acute and chronic medical conditions.

## 8. Conclusions

In a nutshell, platelets are not merely cellular fragments or hemostatic agents but versatile players in the immune system with profound implications for health and many diseases. The ability of these small anucleated blood cells to draw a connection between inflammation and immune responses makes them key targets for therapeutics aimed at thromboinflammation, autoimmunity, and infection. As our understanding of platelet immunobiology continues to evolve, so too will the opportunities to harness their potential for improving patient outcomes in a wide range of immune-mediated conditions.

Despite significant progress in antiplatelet therapy, atherothrombotic events remain a major cause of death worldwide. Current treatments provide incomplete protection and pose bleeding risks. Developing therapies that target platelet receptors and thrombosis pathways while maintaining normal hemostatic function is increasingly important. Continued research is crucial to overcome these challenges and enhance cardiovascular disease management.

## Figures and Tables

**Table 1:** Platelet granules contents.

Alpha Granule Soluble Factors	
Function	Examples
Thrombosis	Fibrinogen, Thrombospondin, Coagulation Factors, Angiostatin, Laminin, Plasminogen
Inflammation	CCL2, CCL3, CCL5, CXCL4, CXL5, CXCL7, CXCL8, CD62P, CD40L, Complement Components (C3a, C5a)
Wound Healing	Endostatin, Thymosin beta 4, VEGF, TGF-beta, PDGF Family
**Alpha Granule Membrane Proteins**	
Protein Type	Examples
Surface Markers	CD9, CD36, CD63, Septin5, Siglec7
Integrins	Integrin αIIbβ3, alpha 6
Receptors	Fc gamma RIIA, CD62P, DC-SIGN,
Membrane Proteins	VAMP 2, 3, 7, 8, Syntaxin 2, Syntaxin 4, 8, 11, Munc18-2, SNAP23
**Dense Granules**	
Component	Examples
Nucleotides	ADP, ATP
Ions	Calcium, Magnesium, Potassium
Small Molecules	Epinephrine, Serotonin, Histamine
Polyphosphates	Polyphosphates, Pyrophosphate
Proteins	STIM1, STIM2
**Lysosome**	
Enzyme	Examples
Glycosidases	α-galactosidase, β-galactosidase
Proteases	Cathepsin A, Hyaluronidase-2 (HYAL2)
Lipases	Glucosylceramidase (GBA), ASAHL
Other Enzymes	Catalase, Lactate Dehydrogenase, Hexosaminidase A/B, Heparinase

**Table 2:** Current and potential antiplatelet therapies.

Molecule	Target	Inhibitory Mechanism
Aspirin and NSAIDs	Cyclooxygenase1	Blocks TXA2 formation
Clopidogrel	P2Y12	Irreversibly inhibits ADP receptors
Ticagrelor	P2Y12	Reversibly inhibits ADP receptors
Cangrelor	P2Y12	Reversibly inhibits ADP receptors
Prasugrel	P2Y12	Irreversibly inhibits ADP receptors
Tirofiban	αIIbβ3	Blocks integrin
Eptifibatide	αIIbβ3	Blocks integrin
Abciximab	αIIbβ3	Blocks integrin
Vorapaxar	PAR1	Blocked thrombin receptors
Iloprost	PGI2 analogue	Increases platelet cAMP levels, thus acting as an intravenous reversible antiplatelet agent
Cilostazol	PDE3A	Inhibits adenosine cellular uptake, increases intraplatelet levels of cyclic AMP
Dipyridamole	PDE3/5	Scavenge peroxy radicals and increase interstitial adenosine levels, increases intraplatelet levels of cyclic AMP
Revacept	CLEC2/GPVI	Competes with platelet GPVI for binding to collagen
Quercetin	PDI	PI3K/Akt inactivation, cAMP elevation

## Abbreviations

The following abbreviations are used in this manuscript:

5-HIAA	5-Hydroxy indoleacetic acid
ADP	Adenosine diphosphate
AG	Alpha Granules
ATP	Adenosine triphosphate
BLOC	Biogenesis of lysosome-related organelles complex
cGAS	Cyclic GMP-AMP synthase
CLRs	C-type lectin receptors
DAPT	Dual antiplatelet therapy
DCs	Dendritic cells
DG	Dense Granules
EGFR	Epidermal growth factor receptor
EPCR	Endothelial protein C receptor
HIT	Heparin-Induced Thrombotic Thrombocytopenia
IVIG	Intravenous immunoglobulin
MK	Megakaryocytes
MVB	Multivesicular bodies
NBEAL2	Neurobeachin-like 2
NET	Neutrophil extracellular traps
NLRs	NOD-like receptors
PAR	Protease-Activated Receptor
PDGF	Platelet-derived growth factor
PF4	Platelet factor 4
PRP	Platelet-rich plasma
PRR	Pattern recognition receptors
SLE	Systemic lupus erythematosus
SNAP23	Synaptosome-associated protein 23
STIM	Stromal Interaction Molecule
STING	Stimulator of interferon genes
TGF-β	Transforming growth factor-beta
TGN	Trans-Golgi network
TLRs	Toll-like receptors
TPO	Thrombopoietin
VAMP	Vesicle-associated membrane protein
VEGF	vascular endothelial growth factor
VITT	Vaccine-induced immune thrombotic thrombocytopenia
VPS16B	Vacuolar Protein Sorting 16B
VPS33B	Vacuolar Protein Sorting 33B
vWF	von Willebrand factor
